# Effect of Different Surface Treatments on the Tensile Bond Strength Between a Heat-Polymerized Silicone-Based Soft Denture Liner and Heat-Cured Denture Base Resin: An In Vitro Study

**DOI:** 10.7759/cureus.112494

**Published:** 2026-07-11

**Authors:** Sumit Verma, Manmeet Gulati, Manmohit Kumar, Mandeep Kumar, Arshjit Kaur Brar, Gurinder Thind

**Affiliations:** 1 Department of Prosthodontics, Desh Bhagat Dental College and Hospital, Mandi Gobindgarh, IND; 2 Department of Prosthodontics, Rayat Bahra Dental College and Hospital, Sahibzada Ajit Singh Nagar, IND

**Keywords:** denture liners, polymethyl methacrylate, sandblasting, surface properties, tensile strength

## Abstract

Introduction: The long-term clinical success of soft denture liners depends on the quality of adhesion between the resilient liner and the denture base resin. Inadequate bonding may result in debonding, microleakage, microbial colonization, and deterioration of the prosthesis performance. The purpose of our study was to assess the effects of various surface treatment techniques on the tensile bond strength of heat-polymerized silicone-based soft denture liners to heat-cured denture base resins.

Materials and methods: This in vitro experimental study included 48 rectangular heat-cured polymethyl methacrylate specimens divided into six groups (n = 8) according to the surface treatment protocol: control, methyl methacrylate (MMA) monomer, phosphoric acid, sandblasting, laser + phosphoric acid, and laser + sandblasting. Following surface treatment, a heat-polymerizing silicone-based soft denture liner was bonded between the acrylic resin blocks. The tensile bond strength was evaluated using a universal testing machine, and the obtained values are expressed in megapascals (MPa). Statistical analysis was performed using one-way analysis of variance (ANOVA) followed by Tukey’s post hoc test, with p < 0.05 considered statistically significant.

Results: The sandblasting group presented the highest mean tensile bond strength (3.80 ± 0.41 MPa), followed by the MMA monomer group (3.45 ± 0.35 MPa) and the laser + sandblasting group (3.33 ± 0.80 MPa). The control group showed the lowest tensile bond strength (2.86 ± 0.44 MPa). A one-way ANOVA showed a statistically significant difference between the study groups (F = 2.746, p = 0.031). Tukey’s post hoc analysis revealed a significant increase in tensile bond strength in the MMA monomer group compared to the control group (p = 0.015). The sandblasting group showed significantly higher bond strength than the control group (p < 0.001), phosphoric acid group (p = 0.012), and laser + phosphoric acid group (p = 0.004).

Conclusion: Surface treatment methods had a significant influence on the tensile bond strength between the heat-polymerizing silicone-based soft denture liner and the heat-cured polymethyl methacrylate denture base resin. Among the evaluated techniques, sandblasting demonstrated the highest tensile bond strength and was found to be the most effective surface treatment method under the conditions of this study.

## Introduction

Soft denture liners are widely used in prosthodontic rehabilitation to improve patient comfort and reduce trauma to underlying oral tissues. These resilient materials act as cushioning interfaces between the denture base and supporting mucosa, thereby distributing occlusal stresses more evenly [1]. Their use is particularly beneficial in patients with severely resorbed ridges, thin mucosa, xerostomia, bruxism, and other conditions associated with compromised denture-bearing tissues [2]. Among the available resilient liners, silicone-based soft liners are preferred because of their superior elasticity, dimensional stability, and long-term resilience [3].

Despite their clinical advantages, one of the major concerns associated with soft denture liners is debonding at the interface between the liner and the denture base resin. Failure of adhesion may result in microleakage, plaque accumulation, fungal colonization, deterioration of mechanical properties, and eventual failure of the prosthesis [4]. Therefore, achieving an adequate bond between the soft liner and polymethyl methacrylate (PMMA) denture base resin is essential for the long-term success of relined dentures [5].

To strengthen the binding between liners and denture base materials, a number of mechanical and chemical surface treatment techniques have been put forth [4]. Surface conditioning procedures, such as monomer application, phosphoric acid etching, airborne-particle abrasion (sandblasting), and laser-assisted treatments, are intended to modify the acrylic resin surface and enhance micromechanical retention or chemical interaction at the bonding interface [6]. Previous studies have reported variable outcomes regarding the effectiveness of these techniques, and no universal consensus has been established regarding the most effective surface treatment protocol [4,6].

Among the available testing methods, tensile bond strength assessment is considered a reliable approach for evaluating the adhesive integrity between the denture base resin and soft lining materials [7]. Therefore, the present in vitro study was undertaken to evaluate the effect of different surface treatment methods on the tensile bond strength between silicone-based soft denture liners and heat-cured PMMA denture base resins. The primary objective was to evaluate and compare the effect of different surface treatment methods on the tensile bond strength between a silicone-based soft denture liner and heat-cured PMMA denture base resin. The null hypothesis was stated as there would be no significant difference in the tensile bond strength between the silicone-based soft denture liner and heat-cured PMMA denture base resin among the different surface treatment methods evaluated.

## Materials and methods

Study design and study setting

This in vitro experimental study was conducted in the Department of Prosthodontics, Desh Bhagat Dental College and Hospital, Mandi Gobindgarh, Punjab, India, from January 2021 to September 2021. The present study was conducted entirely under in vitro laboratory conditions and did not involve human participants, human tissues, patient records, animals, or any identifiable personal data. Therefore, in accordance with institutional ethics guidelines, ethics committee approval was not required. Consequently, the study was granted a waiver of ethical review and informed consent requirements.

Sample size calculation

The sample size was calculated using the G*Power software (version 3.1.9.2; Heinrich-Heine University, Düsseldorf, Germany). A priori power analysis was performed for one-way analysis of variance involving six experimental groups. The minimum necessary sample size was determined to be 48 specimens based on an effect size of 0.54 found in the study by Surapaneni et al. [8], with a statistical power set at 80% and a significance threshold maintained at 0.05. Each group received a total of eight specimens.

Grouping of specimens

**Figure 1:**
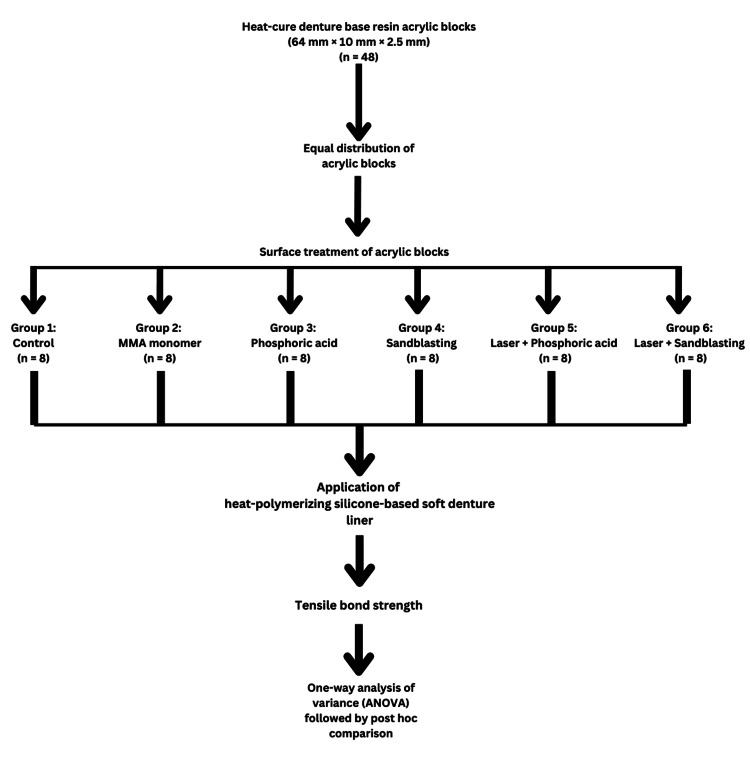
Study flow chart and grouping of samples. MMA: methyl methacrylate. Image Credit: Authors using Canva (Canva Pty Ltd., Sydney, Australia).

A total of 48 specimens were prepared and divided into six groups, with eight samples in each group according to the surface treatment protocol employed. Group I served as the control group without any surface treatment. Group II specimens were treated with methyl methacrylate (MMA) monomers. Group III specimens were treated with 36% phosphoric acid. The Group IV specimens underwent airborne-particle abrasion with alumina particles. Group V specimens underwent laser treatment combined with phosphoric acid application, whereas Group VI specimens underwent laser treatment combined with sandblasting (Figure 1).

Preparation of denture base resin specimens

**Figure 2:**
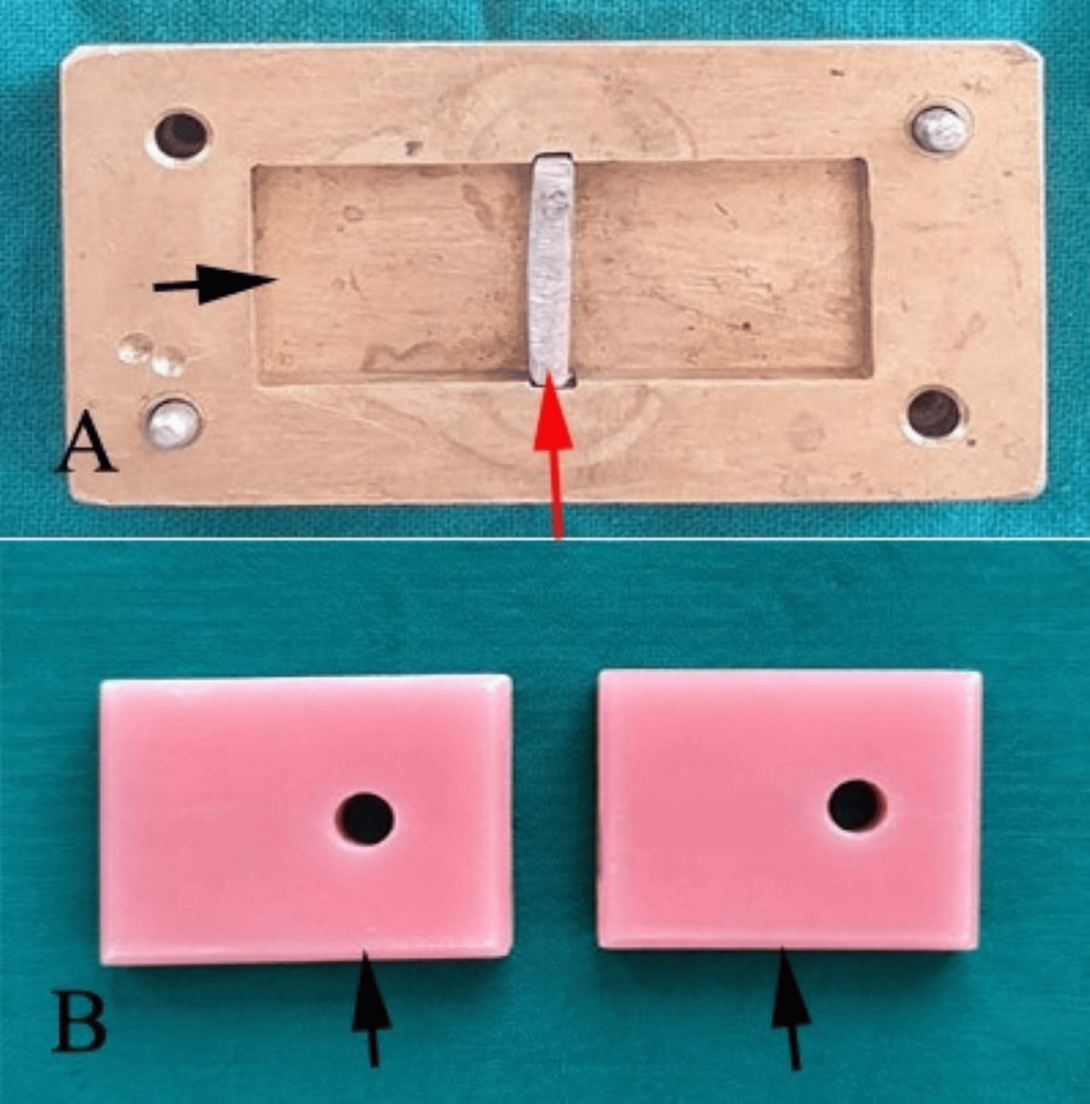
(A) Metal mold for preparing acrylic blocks (black arrow) with a spacer (red arrow) and (B) paired acrylic blocks (black arrows).

Forty-eight rectangular specimens measuring 64 mm × 10 mm × 2.5 mm were fabricated using a customized brass mold with a central spacer measuring 3 mm × 10 mm × 2.5 mm. The spacer created a uniform gap between two acrylic resin blocks, which were later filled with silicone-based soft denture liner material. The heat-polymerized PMMA denture base resin used for specimen fabrication was Dental Products of India (DPI) heat-cure denture base material (Dental Products of India Pvt. Ltd., Mumbai, Maharashtra, India). The polymer and monomer were mixed according to the manufacturer’s recommended weight ratio of 2:1 until homogeneous consistency was achieved. After the material reached the dough stage, it was put into the mold cavity. To ensure appropriate adaption and remove superfluous material, trial closure was carried out. To allow for monomer penetration and even material distribution, the molds were bench-cured for about half an hour (Figure 2).

Polymerization was performed according to the manufacturer’s recommended curing cycle using a conventional acrylization unit. Following polymerization, the specimens were allowed to cool gradually to room temperature prior to deflasking to minimize internal stress and dimensional distortion. The specimens were subsequently trimmed and finished to obtain standardized surfaces.

Surface treatment procedures

The prepared specimens were subjected to different surface treatment protocols, according to their respective study groups. In the control group, no surface treatment was performed and the bonding surfaces were left untreated. In the monomer-treated group, the MMA monomer supplied with the denture base resin system (Dental Products of India Pvt. Ltd., Mumbai, Maharashtra, India) was evenly applied to the bonding surface using a microbrush. The MMA monomer was allowed to remain on the bonding surface for 180 s to facilitate partial dissolution and swelling of the acrylic resin surface, followed by gentle air-drying with oil-free compressed air for 10 s before application of the soft denture liner.

For the phosphoric acid group, the bonding surfaces were conditioned with 36% phosphoric acid gel (Dentsply Sirona, Charlotte, North Carolina, USA). The acid was applied uniformly over the bonding area for the designated period, followed by thorough rinsing with distilled water and air drying to eliminate residual acids and contaminants. About 50 µm aluminum oxide particles, Korox 50 (BEGO Bremer Goldschlägerei Wilh. Herbst GmbH & Co. KG, Bremen, Germany), were used for airborne-particle abrasion in the sandblasting group. To guarantee regular surface roughening, sandblasting was carried out at standardized pressure with the nozzle kept perpendicular to the specimen surface at a given distance. After that, compressed air was used to clean the specimens in order to get rid of any remaining abrasive particles.

In the laser plus phosphoric acid group, the acrylic resin bonding surfaces were irradiated using an Er laser unit (Fotona d.d., Ljubljana, Slovenia) under standardized operating parameters. Laser irradiation was performed at 2,940 nm wavelength, output power of 2 W, frequency of 20 Hz, and pulse energy of 100 mJ. The laser handpiece was maintained perpendicular to the specimen surface at an approximate distance of 10 mm, and the beam was moved uniformly across the bonding area in continuous scanning motion to obtain standardized surface roughening. Following laser irradiation, 36% phosphoric acid gel (Dentsply Sirona, Charlotte, North Carolina, USA) was applied to the treated surface for 30 s. The specimens were then rinsed thoroughly with distilled water and air-dried before the application of silicone-based soft-liner material.

**Figure 3:**
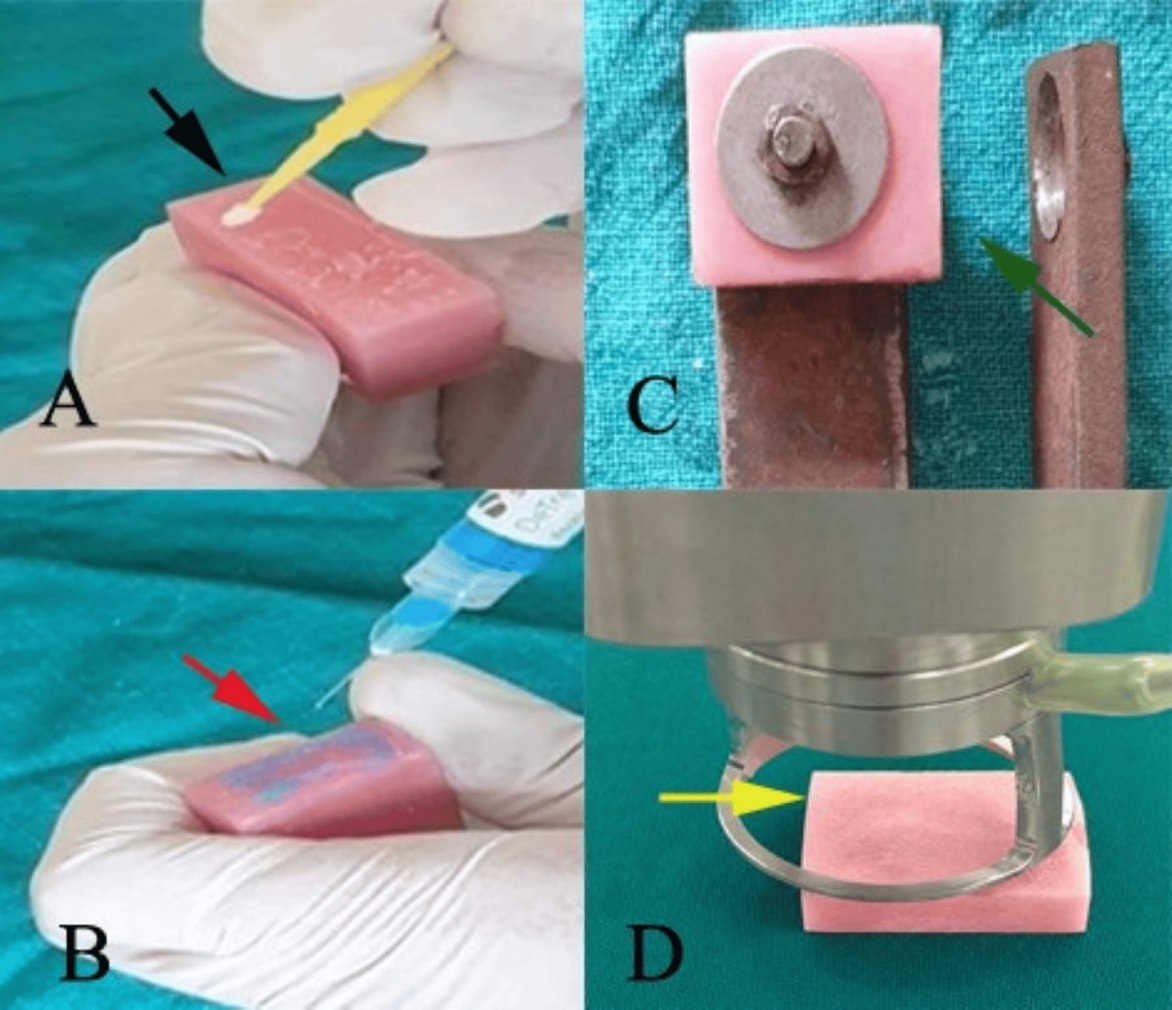
Surface treatment of acrylic blocks with (A) methyl methacrylate monomer (black arrow), (B) phosphoric acid (red arrow), (C) alumina sandblasting (green arrow), and (D) laser treatment (yellow arrow).

In the laser plus sandblasting group, the acrylic resin bonding surfaces were initially treated with Er laser irradiation using the same parameters as described previously. Following laser conditioning, airborne-particle abrasion was carried out using 50 µm aluminum oxide particles, Korox 50 (BEGO Bremer Goldschlägerei Wilh. Herbst GmbH & Co. KG, Bremen, Germany) under a pressure of 2.5 bar for 15 s. The sandblasted nozzle was maintained perpendicular to the bonding surface at a distance of approximately 10 mm to achieve uniform surface roughening and improved micromechanical retention. After completion of the surface treatment, the specimens were cleaned with compressed air to remove residual abrasive particles before the bonding procedures (Figure 3).

Application of silicone-based soft denture liner

Following the completion of the surface treatment procedures, the central spacer region between the acrylic resin blocks was filled with a silicone-based soft denture liner, Molloplast-B (DETAX GmbH & Co. KG, Ettlingen, Germany). To guarantee the full adaptation and removal of voids, the material was carefully packed into the prepared space between the acrylic blocks after being handled in accordance with the manufacturer's instructions. The specimens were then processed according to the recommended curing cycle for the silicone liner material to achieve optimal polymerization and bonding with the denture base resin. After curing, the specimens were allowed to cool gradually and were subsequently finished to remove flash and excess material. Before testing, all specimens were kept at room temperature in distilled water for a full day to stabilize their material qualities.

Evaluation of tensile bond strength

The tensile bond strengths of all specimens were evaluated using a universal testing machine (Paramount Instruments Pvt. Ltd., New Delhi, India). Each specimen was mounted securely in the testing apparatus so that tensile forces were applied along the long axis of the specimen to prevent an uneven stress distribution. A tensile load was applied at a constant crosshead speed until separation occurred between the soft liner and the denture base resin. The maximum load at failure was recorded in Newtons and converted to megapascals (MPa) by dividing the fracture load by the bonding surface area. The obtained values represented the tensile bond strengths of the specimens.

Statistical analysis

Statistical analysis was performed using IBM SPSS Statistics for Windows version 26.0 (IBM Corp., Armonk, New York, USA). Continuous variables were expressed as means and standard deviations, and 95% confidence intervals were calculated for each group. The normality of the data distribution was assessed using the Shapiro-Wilk test. Intergroup comparisons of the tensile bond strength values were performed using one-way analysis of variance (ANOVA). Tukey’s post hoc multiple comparison test was subsequently applied for pairwise comparisons among the groups. Statistical significance was set at p < 0.05.

## Results

The present in vitro study evaluated the effect of different surface treatment methods on the tensile bond strength between silicone-based soft denture liners and heat-cured polymethyl methacrylate denture base resins. A total of 48 specimens were evaluated, and eight were included in each study group. Table 1 presents the descriptive statistics of the tensile bond strength values obtained for the six surface-treatment groups. Among all groups, the sandblasting group demonstrated the highest mean tensile bond strength value (3.80 ± 0.41 MPa), followed by the MMA monomer group (3.45 ± 0.35 MPa) and the laser + sandblasting group (3.33 ± 0.80 MPa). The phosphoric acid group showed a mean tensile bond strength of 3.19 ± 0.35 MPa, whereas the laser + phosphoric acid group demonstrated a mean value of 3.08 ± 0.77 MPa. The control group exhibited the lowest tensile bond strength value (2.86 ± 0.44 MPa). The 95% confidence intervals indicated acceptable precision around the mean estimates, although comparatively wider intervals were observed in the laser-treated groups owing to greater variability among specimens.

**Table 1:** Descriptive statistics of tensile bond strength in megapascals (MPa) between heat-cured polymethyl methacrylate denture base and silicon-based polymethyl methacrylate liner under different surface treatments. The tensile strength has been presented as mean and standard deviation (SD); number of samples in each group has been presented as frequency (n) and percentage (%). Total sample N = 48. CI: confidence interval, MMA: methyl methacrylate.

Group (surface treatment)	Samples, n (%)	95% CI for mean	Tensile bond strength (MPa), Mean ± SD
Control	8 (16.6)	2.49-3.23	2.86 ± 0.44
MMA monomer	8 (16.6)	3.16-3.74	3.45 ± 0.35
Phosphoric acid	8 (16.6)	2.90-3.48	3.19 ± 0.35
Sandblasting	8 (16.6)	3.46-4.14	3.80 ± 0.41
Laser + phosphoric acid	8 (16.6)	2.44-3.72	3.08 ± 0.77
Laser + sandblasting	8 (16.6)	2.66-4.00	3.33 ± 0.80

A comparison of the tensile bond strength values among the six surface treatment groups using a one-way ANOVA is presented in Table 2. ANOVA demonstrated a statistically significant difference among the groups (F = 2.746, p = 0.031), indicating that the type of surface treatment significantly influenced the tensile bond strength between the silicone-based soft liner and heat-cured denture base resin. The calculated effect size (η² = 0.246) suggests a large effect, indicating that approximately 24.6% of the variation in tensile bond strength could be attributed to the different surface treatment methods employed in the study.

**Table 2:** One-way analysis of variance (ANOVA) comparing tensile bond strength across surface treatment groups. *p < 0.05 denotes statistically significant results using one-way ANOVA. df: degree of freedom, η² = eta-squared (effect size).

Source	Sum of square	df	Mean square	F value	p-value	Effect size (η²)
Between groups	2.992	5	0.598	2.746	0.031*	0.246
Within groups	9.153	42	0.218			

Post hoc pairwise comparisons using Tukey’s test are presented in Table 3. The MMA monomer group demonstrated significantly higher tensile bond strength compared with the control group (mean difference = 0.59 MPa, p = 0.015). Similarly, the sandblasting group exhibited significantly greater tensile bond strength than the control group (mean difference = 0.94 MPa, p < 0.001), phosphoric acid group (mean difference = 0.61 MPa, p = 0.012), and laser + phosphoric acid group (mean difference = 0.72 MPa, p = 0.004). No statistically significant differences were observed among the remaining pairwise comparisons (p > 0.05).

**Table 3:** Post hoc Tukey’s test for pairwise comparisons of tensile bond strength between surface treatment groups. *p < 0.05 denotes statistical significance using Tukey’s HSD post hoc test. Positive mean difference denotes higher bond strength in the first group. CI: confidence interval, MMA: methyl methacrylate, HSD: honestly significant difference.

Comparison	Mean difference (MPa)	q statistic	p-value	95% CI
Control vs MMA monomer	-0.59	2.53	0.015*	-1.06 to -0.12
Control vs Phosphoric acid	-0.33	1.41	0.165	-0.80 to 0.14
Control vs Sandblasting	-0.94	4.03	<0.001*	-1.41 to -0.47
Control vs Laser + phosphoric acid	-0.22	0.94	0.351	-0.69 to 0.25
Control vs Laser + sandblasting	-0.47	2.01	0.050	-0.94 to 0.00
MMA monomer vs Phosphoric acid	0.26	1.11	0.272	-0.21 to 0.73
MMA monomer vs Sandblasting	-0.35	1.50	0.141	-0.82 to 0.12
MMA monomer vs Laser + phosphoric acid	0.37	1.59	0.120	-0.10 to 0.84
MMA monomer vs Laser + sandblasting	0.12	0.51	0.610	-0.35 to 0.59
Phosphoric acid vs Sandblasting	-0.61	2.61	0.012*	-1.08 to -0.14
Phosphoric acid vs Laser + phosphoric acid	0.11	0.47	0.640	-0.36 to 0.58
Phosphoric acid vs Laser + sandblasting	-0.14	0.60	0.552	-0.61 to 0.33
Sandblasting vs Laser + phosphoric acid	0.72	3.08	0.004*	0.25 to 1.19
Sandblasting vs Laser + sandblasting	0.47	2.01	0.050	0.00 to 0.94
Laser + phosphoric acid vs Laser + sandblasting	-0.25	1.07	0.290	-0.72 to 0.22

Overall, the findings of the present study demonstrated that surface treatment procedures improved the tensile bond strength between the silicone-based soft denture liner and heat-cured PMMA denture base resin when compared with the untreated control group. Among the evaluated surface treatment methods, sandblasting produced the highest tensile bond strength and was found to be the most effective surface conditioning procedure under the conditions of this study.

## Discussion

The degree of adhesion between the soft liner material and the denture base resin is a major factor in the long-term clinical effectiveness of resilient denture liners. Debonding, microleakage, microbial colonization, discoloration, deterioration of mechanical qualities, and ultimately prosthesis failure can result from bonding failure [5,6]. Therefore, strengthening the binding between polymethyl methacrylate denture base resin and soft denture liners continues to be a crucial field of prosthodontics research. The current in vitro study assessed how various surface treatment techniques affected the tensile bond strength between heat-cured polymethyl methacrylate denture base resins and silicone-based soft denture liners.

In the present study, the sandblasting group demonstrated the highest mean tensile bond strength among all evaluated groups and showed statistically significant superiority over the control, phosphoric acid, and laser + phosphoric acid groups. The enhanced bond strength observed after airborne-particle abrasion can be attributed to the increase in surface roughness and surface area produced by aluminum oxide particles, which improves the micromechanical interlocking between the denture base resin and the soft liner material. Sandblasting also enhances surface wettability and facilitates mechanical retention at the bonding interface [9].

The systematic evaluation by Hamedirad et al. [10], which found that sandblasting did not considerably increase the tensile bond strength of silicone-based soft liners, contrasts with the current findings. In contrast to the control group, their subgroup analysis revealed that blasting pressures higher than 1 bar considerably strengthened the binding. Therefore, the use of standardized sandblasting techniques and controlled abrasive pressure may have produced more surface roughness and improved micromechanical retention between the silicone liner and denture base resin, which may be responsible for the improved results seen in this study.

Our findings are in agreement with studies by Kuźniarski et al. [11] and Akin et al. [12], who demonstrated that airborne-particle abrasion with aluminum oxide particles enhanced the bond strength between silicone-based liners and PMMA denture base resin. Gopal et al. [13] also observed improved bond strength following mechanical surface-roughening procedures, such as sandpaper abrasion and surface perforation. These studies support the concept that increased surface irregularities promote stronger adhesion via improved mechanical retention.

The MMA monomer-treated group in the present study also demonstrated significantly greater tensile bond strength than the untreated control group. Application of the MMA monomer likely causes partial dissolution and swelling of the PMMA surface, resulting in enhanced diffusion and interpenetration between the denture base resin and liner material. This chemical interaction contributes to improved adhesion and stronger interfacial bonding [6].

The present results are consistent with the findings of Surapaneni et al. [8], who reported superior bond strength following MMA surface treatment compared to other conditioning methods. Similarly, Sarac et al. [14] concluded that monomer treatment effectively reduced microleakage and improved the bond strength between resilient liners and acrylic resins. These findings collectively support the beneficial effect of monomer surface conditioning in enhancing chemical bonding at the liner-resin interface.

When compared to the control group, the tensile bond strength of the phosphoric acid-treated specimens in this investigation improved somewhat, although the difference was not statistically significant. By increasing the surface energy and creating superficial surface imperfections, phosphoric acid conditioning may encourage adhesion. However, the impact seems to be less noticeable than that of MMA therapy and airborne particle abrasion. Similar results were reported by Gundogdu et al. [15], who discovered that acid etching resulted in bond strength values that were similar to the control group's without significantly improving.

The laser-assisted surface treatment groups in the present study did not demonstrate a significant improvement in tensile bond strength compared with the conventional surface treatment methods. Although laser irradiation can produce surface roughness and microstructural alterations, the combined laser protocols evaluated in the present study failed to provide superior adhesion. One possible explanation may be that excessive surface irregularities created by the laser treatment interfere with the intimate adaptation of the liner material to the acrylic surface. Additionally, the thermal effects generated during laser irradiation may alter the chemical composition and surface integrity of the polymethyl methacrylate resin.

The present findings are partially consistent with a study conducted by Usumez et al. [16], who reported that laser treatment produced surface texture changes without significantly improving the bond strength. The findings of the present study regarding laser-assisted surface treatment are in contrast with the systematic reviews conducted by Özdemir and Özdoğan [17] and Alhamdan [18], who reported that laser surface treatment may improve the bond strength between resilient liners and polymethyl methacrylate denture base resin. The discrepancy between the present findings and these reviews may be attributed to differences in the laser type, wavelength, energy settings, irradiation duration, and liner materials evaluated in various studies. Excessive surface alterations or thermal effects produced by laser irradiation may adversely affect the bonding interface, which could explain the comparatively low bond strength values observed in the present study. Therefore, the effectiveness of laser-assisted conditioning appears to depend heavily on the specific parameters used during treatment.

The control group demonstrated the lowest tensile bond strength values among all the study groups, indicating that untreated PMMA surfaces provide inadequate bonding conditions for silicone-based soft liners. This finding highlights the importance of surface modification before the application of resilient liner materials. The findings of this study have several important clinical implications. Adequate bonding between soft liners and denture base resins is essential for maintaining the durability, hygiene, and functional performance of relined dentures. Surface treatment methods, such as sandblasting and MMA monomer conditioning, may be incorporated clinically to improve liner adhesion and reduce the risk of debonding and microbial colonization. Improved bond strength may ultimately enhance patient comfort, prosthesis longevity, and overall treatment success in patients who require resilient denture liners.

Despite these significant findings, the present study has several limitations. First, the investigation was conducted under in vitro conditions, which cannot fully replicate the complex oral environment characterized by thermal fluctuations, cyclic masticatory loading, salivary enzymes, and microbial activity. Second, the study evaluated the immediate bond strength only, without incorporating artificial aging protocols such as thermocycling or prolonged water storage; therefore, the long-term durability of the bond could not be assessed. Additionally, failure mode analysis was not performed, limiting the characterization of the adhesive interface following debonding. Furthermore, only one silicone-based soft denture liner and one heat-cured polymethyl methacrylate denture base resin were evaluated, which may limit the generalizability of the findings to other commercially available materials. Future studies should incorporate artificial aging procedures, failure mode analysis, different liner and denture base materials, and varied laser parameters, as well as long-term in vivo investigations, to further validate the clinical applicability of these findings.

## Conclusions

Within the limitations of this in vitro study, surface treatment methods significantly influenced the tensile bond strength between the silicone-based soft denture liner and heat-cured polymethyl methacrylate denture base resin. Among the evaluated surface treatment protocols, sandblasting produced the highest tensile bond strength and was the most effective method for enhancing adhesion. MMA monomer conditioning also significantly improved bond strength compared with the untreated control. In contrast, the laser-assisted surface treatment protocols evaluated in this study did not demonstrate superior bonding performance under the tested conditions. Further studies incorporating artificial aging procedures and long-term clinical evaluation are warranted to confirm the durability and clinical applicability of these findings.
